# Neurotoxicity of Combined Exposure to the Heavy Metals (Pb and As) in Zebrafish (*Danio rerio*)

**DOI:** 10.3390/toxics12040282

**Published:** 2024-04-11

**Authors:** Ming Liu, Ping Deng, Guangyu Li, Haoling Liu, Junli Zuo, Wenwen Cui, Huixian Zhang, Xin Chen, Jingjing Yao, Xitian Peng, Lijun Peng, Jiao Liu, Wenting Zheng, Wei Yan, Ning Luan

**Affiliations:** 1 College of Fisheries, Huazhong Agricultural University, Wuhan 430070, China; liuming.1212@163.com (M.L.); liguangyu@mail.hzau.edu.cn (G.L.); liuhaoling@webmail.hzau.edu.cn (H.L.); zuojl1005@163.com (J.Z.); zheng_teiko@hotmail.com (W.Z.); 2 Wuhan Academy of Agricultural Sciences, Wuhan 430056, China; dp@wuhanagri.com; 3 Hubei Key Laboratory of Nutritional Quality and Safety of Agro-Products, Institute of Quality Standard and Testing Technology for Agro-Products, Hubei Academy of Agricultural Sciences, Wuhan 430064, China; wenwencui.305@163.com (W.C.); zhanghuixianto@163.com (H.Z.); hgchenxin@163.com (X.C.); yyy0779@163.com (J.Y.); pxitian@aliyun.com (X.P.); penglijun@hbaas.com (L.P.); zhibiaosuo204@163.com (J.L.)

**Keywords:** zebrafish, heavy metals, co-exposure, neurotoxicity, neurotransmitter, HPI axis

## Abstract

Lead (Pb) and arsenic (As) are commonly occurring heavy metals in the environment and produce detrimental impacts on the central nervous system. Although they have both been indicated to exhibit neurotoxic properties, it is not known if they have joint effects, and their mechanisms of action are likewise unknown. In this study, zebrafish were exposed to different concentrations of Pb (40 μg/L, 4 mg/L), As (32 μg/L, 3.2 mg/L) and their combinations (40 μg/L + 32 μg/L, 4 mg/L + 3.2 mg/L) for 30 days. The histopathological analyses showed significant brain damage characterized by glial scar formation and ventricular enlargement in all exposed groups. In addition, either Pb or As staining inhibited the swimming speed of zebrafish, which was enhanced by their high concentrations in a mixture. To elucidate the underlying mechanisms, we examined changes in acetylcholinesterase (AChE) activity, neurotransmitter (dopamine, 5-hydroxytryptamine) levels, HPI axis-related hormone (cortisol and epinephrine) contents and neurodevelopment-related gene expression in zebrafish brain. The observations suggest that combined exposure to Pb and As can cause abnormalities in swimming behavior and ultimately exacerbate neurotoxicity in zebrafish by interfering with the cholinergic system, dopamine and 5-hydroxytryptamine signaling, HPI axis function as well as neuronal development. This study provides an important theoretical basis for the mixed exposure of heavy metals and their toxicity to aquatic organisms.

## 1. Introduction

The environment is being contaminated by a wide range of pollutants, including heavy metals. As a result, the health consequences of them are attracting the attention of the public and health experts worldwide [1]. Although several of the adverse health effects of heavy metals have been known for a long time, exposure continues and is even increasing in some regions of the world [2,3]. Among these, Pb and As are of concern as environmental contaminants and potential neurotoxicological hazards [3,4]. Pb is widely occurring in plastics, batteries, water pipes, paints, pesticides and leaded petrol [5]. The concentration of Pb in uncontaminated groundwater ranges from 1 µg/L to 60 µg/L. When its levels reach 0.1 mg/L, the self-purification of water bodies can be inhibited [6]. In view of its persistence and poor degradation rate, Pb is poisonous, even at a low dose, due to its accumulation. Pb from food and water is absorbed in the gastrointestinal (GI) tract and distributed to several organs [7], where 3–10% (adult) of the oral dose of water-soluble Pb can be absorbed and may cause neurological, blood and nephrotoxic effects [8], thereby constituting an underlying hazard to public health [9]. Pb is a well-known neurotoxin that can inflict damage on the central nervous system. Acute Pb exposure can contribute to neurotoxic effects in experimental animals like scleractinian fish and rats, such as aberrant behavior, learning disabilities and impaired cognitive function [10,11]. The neurotoxic effects of Pb at 10 and 100 μM have been investigated, and it was demonstrated that they are induced by a variety of mechanisms, including the disruption of neurotransmitters [12], alterations in the gene expression of the central nervous system [13], the disruption of the function of the dopamine and cholinergic systems [14,15], the apoptosis and disruption of the hypothalamic–pituitary–adrenal axis [16]. Equally important, reported zebrafish embryos exposed to 0.2 mM of Pb for 24, 48 and 72 hpf exhibit significant neurotoxic symptoms in the form of delayed swimming movements [17]. Pb (0.2 mM)-treated zebrafish embryos for 24, 48 and 72 hpf exhibit alterations in neural compartment formation, hindbrain branchial motor neurons and neurovascular structures [18]. Consequently, the nervous system can be considered a potential target for Pb.

As is commonly a by-product of smelting, fossil fuel combustion and pesticide production [19,20]. Over the past few years, As pollution has significantly increased in Asia and the Americas [21]. Aquatic habitats, including freshwater habitats, are frequently impacted by As contamination, as it can be found in groundwater and surface water in quantities exceeding the World Health Organization’s worldwide recommendation of 0.01 mg/L. As levels have reached 5.1–2.4 mg/L in Los Azufres, Michoacan, and 419.8 µg/L in Mexico [22,23]. Fish can have up to 10 μg of As per gram of dry weight, whereas terrestrial species can have 30 ng of As per gram on a dry basis [24]. The elevated capacity of fish to uptake As may render it more susceptible to this contaminant in the environment. In freshwater systems, the massive bioaccumulation of As through the trophic chain impacts human health via the consumption of aquatic species from contaminated sites. As is mainly deposited in the liver, kidneys and brain, triggering renal and neurological effects [25]. Mounting evidence supports As exposure resulting in neurotoxicity, particularly cognitive and neurobehavioral dysfunction [26,27]. Research has shown that the mechanisms of As-induced neurotoxicity in the brain are tightly linked to neurotransmitter transmission [28] and the regulation of the glucocorticoid system [29], which together can account for behavioral impairment. Tadanobu et al. reported changes in monoamine metabolism and motor activity in the mouse brain. They discovered that As can pass across the blood–brain barrier, then enter the brain in minute amounts, with consequences for metabolism [30]. As also influenced the synthesis of brain monoamines and caused behavioral abnormalities in mice. It has already been shown that prolonged exposure to environmentally relevant doses of As produces a wide range of neurobehavioral changes in zebrafish during development [31], causing multiple alterations in the dopamine system [32] and disrupting the endocrine system [33]. In summary, the effects of As on the nervous system are not negligible.

Heavy metals tend to co-occur in the natural environment [34], so investigating single metal exposures may not adequately predict health risks. Antagonistic or synergistic interactions between various metals in biological samples were extensively described [35]. Fowler et al. demonstrated that toxic metal interactions altered toxicity in rats after 10–13 weeks of simultaneous exposure to lead, cadmium and arsenic [36]. Recent years have seen an influx of investigations into the mechanisms of heavy metal interactions. Agrawal et al. discovered that joint lead and cadmium exposure exacerbated neurotoxicity in rats by modulating the expression of histone deacetylases [14]. De León et al. found that chronic exposure to lead and copper in water exacerbated metal-independent neurotoxic effects on the dopaminergic and the 5-hydroxytryptaminergic systems of zebrafish [14,15]. It has been shown that brain biogenic amines and acetylcholinesterase were altered in the tissues of rats poisoned with the combination of lead, arsenic and mercury. To date, a combination of Pb and As exposure has been identified as causing anxiety-like behavior and impairing spatial memory and learning in mice [37]. Although relevant studies on the compound toxic effects of Pb and As on zebrafish development were carried out [38,39], there is still much room for the exploration of other toxicities. On the other hand, since the nervous system is more sensitive to heavy metal pollutants compared to other organs, single heavy metals all show strong neurotoxicity [40]. However, it is unclear whether Pb and As exposure leads to neurological damage in zebrafish and whether concurrent exposure exacerbates the toxic effects. Given the widespread distribution of Pb and As and the existence of common routes of neurotoxicity, it is essential to clarify the neurotoxicological profile of their combined effects and to probe the exact mechanisms.

In this context, we designed this experiment using zebrafish as a model to elucidate the toxic effects of long-term exposure to Pb, As and their combinations on the nervous system of adult zebrafish. Histopathological analyses of the zebrafish brain were carried out after 30 days of exposure. Zebrafish swimming speed was monitored. AChE activity and levels of dopamine (DA) and pentraxin (5-HT) were also assayed in zebrafish brains, along with levels of cortisol (Cor) and adrenaline (EPI). The transcript levels of neurotransmitter pathways, neuronal development-related and hypothalamicpituitary-interrenal (HPI) axis-related genes (*shha*, *gfap*, *syn2a*, *pcdh18b*, *crh*, *acth*, *nr4a2b*, *manf*, *ache*, *gap43* and *elavl3*) were also further examined. This study evaluated the individual and combined toxic effects of Pb and As and will significantly enhance our insights into the combined effects and mechanisms of Pb and As in zebrafish neurotoxicity.

## 2. Materials and Methods

### 2.1. Chemicals and Test Fish

Lead acetate ([(CHCOO)zPb·3HO], Catalog #10012416) was purchased from Sinopharm Shanghai Chemical Reagent Company (Shanghai, China). Sodium arsenite (NaAsOz, Catalog #A25410) was obtained from Beijing Innochem Technology Co., Ltd. (Beijing, China). All the other chemicals were of analytical grade. Moreover, 4-month-old adult zebrafish (AB line) were purchased from Shanghai Fish Biology Co., Ltd. (Shanghai, China). All experiments on zebrafish followed the regulations of the Institutional Animal and Care Use Committee of Huazhong Agricultural University (Ethical number, HZAUFI-2023-0011, IACUC, Wuhan, China) and were conducted at the Laboratory Animal Center of Huazhong Agricultural University.

### 2.2. Adult Zebrafish Culture

At the beginning of the exposure experiment, the purchased zebrafish were temporarily cultivated in the aquarium for two weeks. They were cultivated at 28 °C for about 14:10 h in a light–dark cycle. During this period, newly hatched salt shrimp (*Artemia salina*) were fed two times daily. Waste and residues were removed daily.

The experiment was set up with 7 exposure concentrations: control, 40 μg/L Pb, 4 mg/L Pb, 32 μg/L As, 3.2 mg/L As, 40 μg/L Pb + 32 μg/L As and 4 mg/L Pb + 3.2 mg/L As. The duration of exposure was 30 days. Each concentration group contained 3 replicates in which 20 healthy females and 20 healthy males were placed. In this study, the exposures were semi-static. Concentrations of exposure to the heavy metals Pb and As were based on environmental concentrations and semi-lethal concentrations from prior research [41,42,43]. Half of the exposure solution was replaced every three days to preserve the stationary levels (Tables S2 and S3). Following 30 days of exposure, zebrafish were anesthetized with 0.02% tricaine methane sulfonate (MS-222, Sigma-Aldrich, Darmstadt, Germany). Brain tissue was autopsied in ice to serve for repeat samples.

### 2.3. Histopathological Analysis

The brain tissue (n = 3) of zebrafish was surgically amputated on ice and fixed in 4% paraformaldehyde for 24 h. Brain samples were then dehydrated in various concentrations of ethanol and embedded in paraffin. The sagittal plane (4 μm) was stained with hematoxylin–eosin (H&E). Histopathological evaluation was performed with a somatic view microscope (LeicaM205FA, Deerfield, IL, USA).

### 2.4. Swimming Behavior Detection

The locomotor activity of zebrafish was quantified using the Viewpoint Life Sciences visual tracking system. After 30 days of Pb and As exposure, female and male zebrafish (n = 10) were randomly selected from each group to test their locomotor ability separately. Zebrafish were individually placed in a tank (10 cm × 12.5 cm × 15 cm, height × width × length), and their swimming behavior was recorded. Every test time was 10 min. At end of all examinations, the data were analyzed using software (Stoelting Co., Shanghai, China).

### 2.5. AChE Activity Assay

First, weighed brain tissue samples (each replicate contains 5 brains, n = 3) were placed in plastic centrifuge tubes. Depending on the ratio of weight (g) to volume (mL), saline (0.9% NaCl) was added to the centrifuge tubes to dilute the samples into a 10% tissue homogenate, then homogenize and centrifuge for 10 min (4 °C, 2500 rpm). The supernatant was transferred to a new tube, and enzyme activity was measured using an AChE assay kit (Nanjing Jiancheng Institute of Biological Engineering, Nanjing, China) according to the manufacturer’s instructions. Light density was recorded at 412 nm. Protein concentration was determined by the BCA method using bovine serum albumin (BSA) as a standard at 595 nm.

### 2.6. Determination of Neurotransmitter Levels

Neurotransmitter levels, including DA and 5-HT levels, were determined by the corresponding ELISA kits (Nanjing Jiancheng Institute of Biological Engineering, Nanjing, China) according to the manufacturer’s protocol. To start with, brain tissue samples (each replicate contains 5 brains, n = 3) were weighed, diluted to 10% (*w*/*v*) with PBS, homogenized and then centrifuged at 4 °C for 20 min to collect the supernatant. After adding samples, washing the plate, incubating and stopping the reaction, the absorbance at 450 nm was detected by enzyme marker. Lastly, the content of DA and 5-HT in the brain tissue of each treatment group was calculated using the standard curve.

### 2.7. HPI Axis-Related Hormone Content Determination

Blood samples from 10 zebrafish of the same sex were collected together as a replicate (n = 3). The levels of fish Cor and EPI were determined using ELISA kits (Nanjing Jiancheng Institute of Biological Engineering, Nanjing, China) according to the manufacturer’s protocol. Samples were weighed and first diluted in PBS at a ratio of 1:9 (*v*/*v*) for tissue homogenization. Then, centrifugation at 3000× *g* for 20 min was performed to collect the supernatant for assays. The absorbance was measured at 450 nm using an enzyme marker and the concentrations of zebrafish Cor and EPI were calculated from the standard curve.

### 2.8. Quantitative Real-Time PCR (qPCR) Assay

Based on earlier studies, Trizol (Takara, Kusatsu, Japan) was obtained and prepared for the extraction of RNA. The integrity of the extracted RNA and its purity were examined using agarose gel electrophoresis, with the OD260/OD280 ratio used to evaluate the quality and quantity of RNA. RNA treated with DNA ase I was reverse transcribed to cDNA with the Prime Scrip™ RT kit (Perfect Real Time, Takara, Japan) using the thermocycler (Thermo Fisher, Waltham, MA, USA). Real-time quantitation polymerase chain reaction was performed using the SYBR PreMix Ex Taq™ kit (containing SYBR PreMix Ex Taq™ and ROX reference dye (Takara, Japan)). Primer 3 software (http://frodo.wi.Mit.edu) (accessed on 15 June 2023) (Table S1) was utilized to design gene-specific primers related to neurotransmitter pathways, as well as neuronal development-related and HPI axis-related genes. QRT-PCR parameters were comparable to previous studies [44]. *β-actin* was selected as the internal reference, and gene expression data were analyzed according to the 2^−ΔΔCt^ method of Livak and Schmittgen [45].

### 2.9. Statistical Analysis

The data obtained were statistically analyzed by the software SPSS 26.0 (Chicago, IL, USA) and graphics were indicated using GraphPad Prism 8 (San Diego, CA, USA). Kolmogorov–Smirnov test was first used to test the data for normal distribution, and Levene test was used to test the homogeneity of variance. Then, one-way ANOVA following Tukey’s test was used to determine if the treatment results were significantly different from the control (*p* < 0.05). There were three independent replications of the test, and the values were expressed as mean ± standard error (mean ± SEM).

## 3. Results

### 3.1. Histopathology Analysis

**Figure 1:**
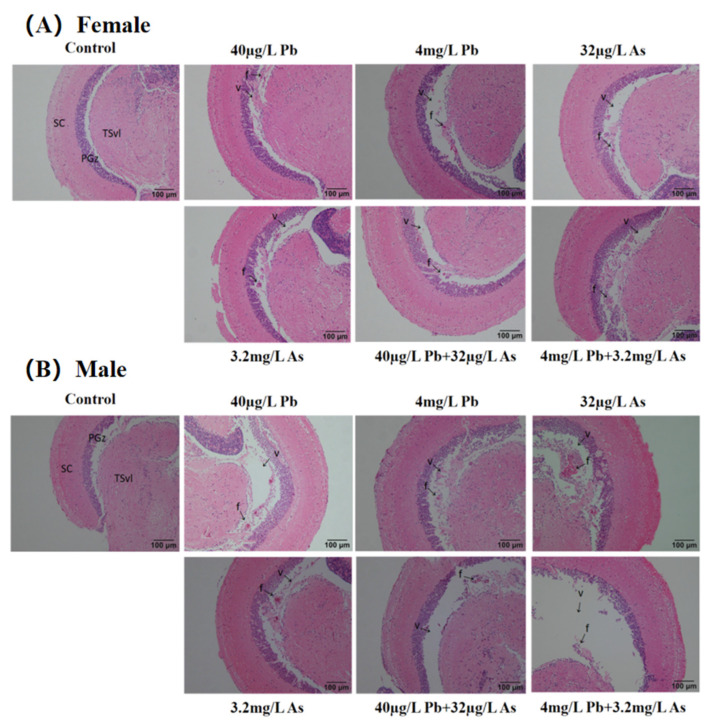
Histopathological analysis of female (**A**) and male (**B**) zebrafish brain (scale bar of 100 µm). Note: SC is limbic layer; PGZ is periaqueductal gray matter zone; TSv1 is semicircular ring ventral lateral nucleus; f is glial scar formation; v is ventricular enlargement.

All zebrafish had histopathological damage to the brain from heavy metal exposure (Figure 1). In terms of H&E staining, neuroglial scar formation and ventricular enlargement were visible in each group except the control group. In addition, ventricular enlargement was more pronounced in zebrafish in the high concentrations of the combined Pb and As exposure group.

### 3.2. Swimming Performance Analysis

**Figure 2:**
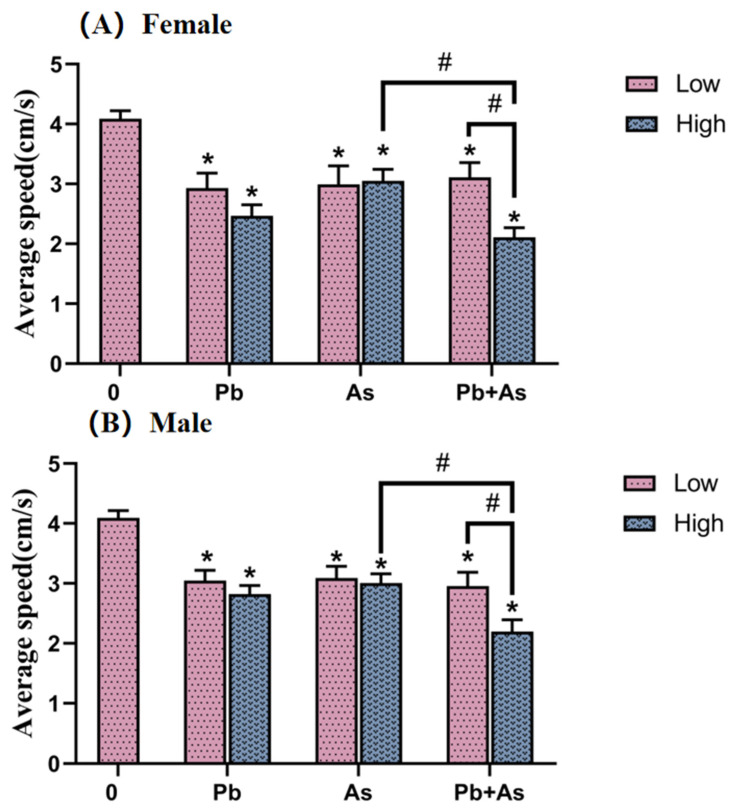
Swimming behaviors of female (**A**) and male (**B**) zebrafish after 30 days of metal exposure (mean ± SEM, n = 10). * indicate significant differences between the treatment and control groups (*p* < 0.05), while # are considered to be significant differences between the two different heavy metal treatment groups (*p* < 0.05).

The swimming ability of zebrafish was detected and then analyzed (Figure 2). We observed an equal tendency of changes in the swimming behavior of females and males with different heavy metal treatments. The average swimming speed of zebrafish was statistically significantly lower in all heavy metal-exposed groups compared to the control group. There was a significant difference between the high-concentration Pb + As exposure group and the low co-exposure group. In the high-concentration treatment group, Pb + As co-exposure showed a further reduction in swimming speed than the As single-exposure group.

### 3.3. AChE Activity of Brains

**Figure 3:**
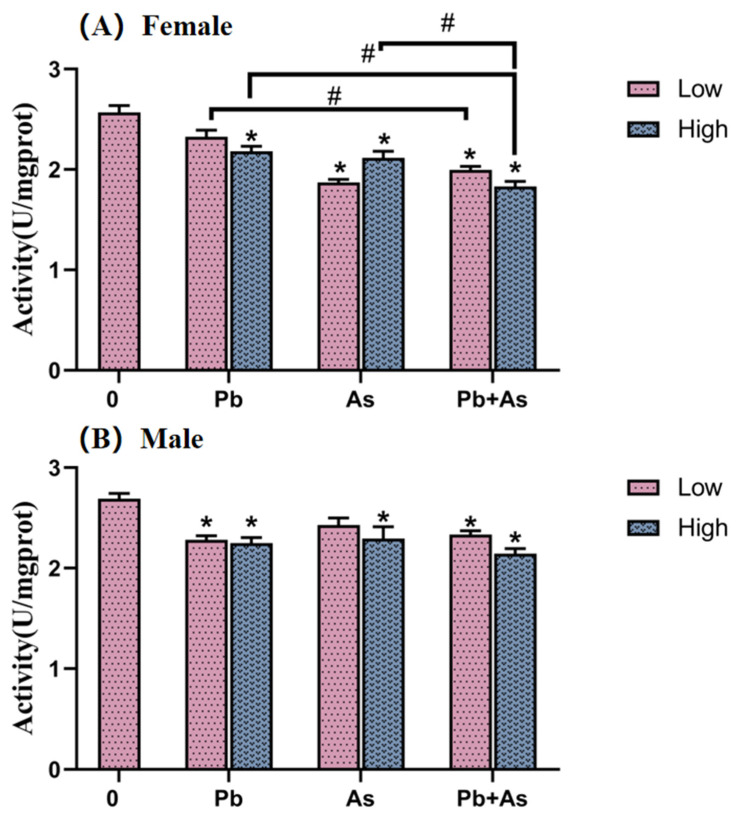
AChE activity in female (**A**) and male (**B**) zebrafish brain after 30 days of As and Pb exposure (mean ± SEM, n = 3). * indicate significant differences between the treatment and control groups (*p* < 0.05), while # are considered to be significant differences between the two different heavy metal treatment groups (*p* < 0.05).

In female and male zebrafish, AChE activity was monitored in brain tissue, as illustrated in Figure 3. As for female zebrafish, exposure to As, Pb + As and high concentrations of Pb resulted in a significant decrease in AChE enzyme activity. AChE levels were remarkably inhibited in the Pb + As low-concentration treatment group compared to the Pb low-concentration single exposure group. Moreover, there were also statistically significant differences in the effects of heavy metal exposure alone and combined exposure on AChE enzyme activity in the high-concentration treatment group. In the male zebrafish brain, AChE enzyme activity was significantly decreased in the Pb, high-concentration As and Pb + As groups compared to the control group.

### 3.4. DA and 5-HT Contents in Brains

**Figure 4:**
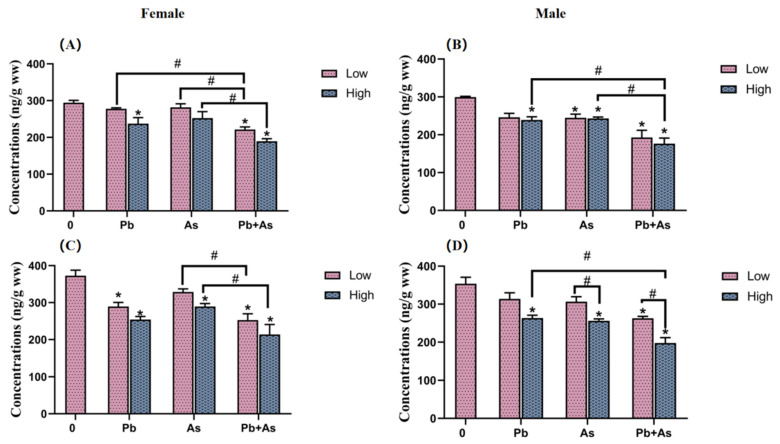
DA (**A**,**B**) and 5-HT (**C**,**D**) content in the brains of female and male zebrafish after 30 days of exposure (mean ± SEM, n = 3). * indicate significant differences between the treatment and control groups (*p* < 0.05), while # are considered to be significant differences between the two different heavy metal treatment groups (*p* < 0.05).

To identify the influence of exposure to different concentrations of Pb, As and their mixtures on neurotransmitter levels in zebrafish, we measured the amounts of DA and 5-HT in the brains of both female and male zebrafish. DA content was barely affected by exposure groups in female fish brain tissue, except for the high Pb concentration and combined exposure groups (Figure 4). In the low-concentration treatment group, there was a significant difference in the effect of heavy metal exposure alone versus combined exposure on DA levels. DA levels were further reduced in the joint group compared with the high-concentration As exposure group. Also, 5-HT levels were found to be significantly lower in the Pb and high-concentration As and Pb + As groups. Both As exposure singly along with combined exposure showed marked variations in the impacts on 5-HT. In male fish brain tissue, exposure to As and high concentrations of Pb and Pb + As caused a significant decrease in DA content. The DA level was further reduced in the high-concentration combined exposure group than the group exposed to heavy metals alone. Concurrently, high concentrations of Pb, As and the combined exposure groups all led to a distinct drop in 5-HT levels; there were prominent differences in 5-HT levels between the low- and high-concentration treatment groups of As and Pb + As. Among the high-concentration exposure groups, there was a considerable variance in the effect of Pb exposure alone versus combined exposure on DA levels.

### 3.5. HPI Axis Hormone Content

**Figure 5:**
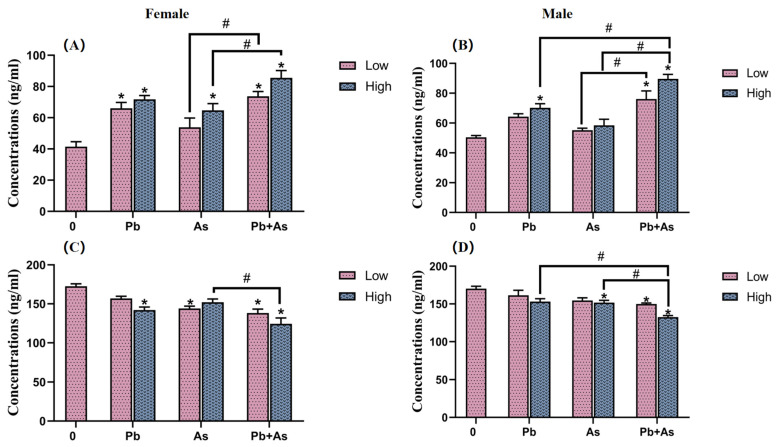
Cortisol (**A**,**B**) and EPI (**C**,**D**) levels in female and male zebrafish after 30 days of exposure (mean ± SEM, n = 3). * indicate significant differences between the treatment and control groups (*p* < 0.05), while # are considered to be significant differences between the two different heavy metal treatment groups (*p* < 0.05).

Cor and EPI levels within female and male zebrafish were also modified, as illustrated by Figure 5. Exposure to Pb, high concentrations of As and combined exposure significantly elevated Cor levels in female zebrafish. Also, male zebrafish with high Pb exposure group and Pb + As exposure group remarkably raised Cor levels. The impact of As exposure alone and combined exposure on Cor showed significant differences in both sexes. Moreover, Cor levels were further increased in the high combined exposure group in male zebrafish compared to the high-Pb exposure group. In female zebrafish, EPI levels were significantly lower in the high-Pb concentration, low-As concentration and Pb + As groups. Distinctly lower EPI levels were also observed in male zebrafish in the high-concentration As and Pb + As groups. In the high-exposure group, both As exposure only and combined exposure showed significant differences in the changes in EPI levels in zebrafish. The EPI levels were even lower in the joint exposure group of male zebrafish compared with Pb exposure alone.

### 3.6. Alterations in Gene Transcript Levels

**Figure 6:**
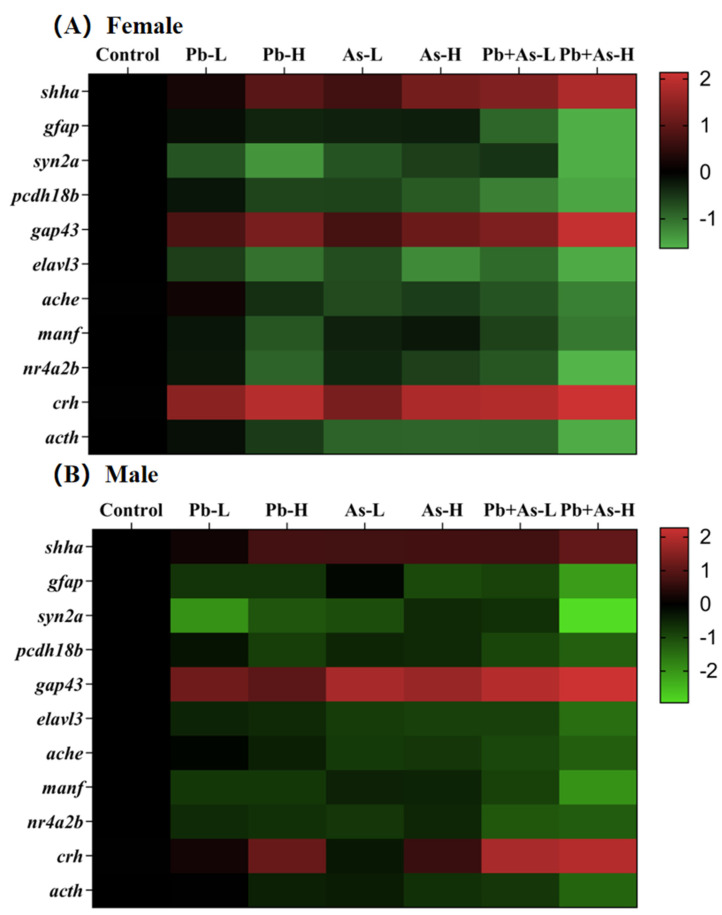
The heat map of gene expression related to neurotransmitter pathways, neuronal development and HPI axis in the brains of female (**A**) and male (**B**) zebrafish after treatment with heavy metals (mean ± SEM, n = 3).

The expression levels of genes related to neurotransmitter pathways, neuronal development and HPI axis were measured post-exposure, and the results are presented in Figure 6. These genes consisted of *shha*, *gfap*, *syn2a*, *pcdh18b*, *gap43*, *elavl3*, *ache*, *manf*, *nr4a2b*, *crh* and *acth*. Based on the results of the heat map, we found that long-term exposure to heavy metals (Pb, As) had essentially the same gene expression trends in zebrafish of different sexes. In zebrafish brains, the expression of *shha*, *gap43* and *crh* were significantly upregulated, while the levels of other genes were downregulated significantly compared to the control group. In female zebrafish brains, the low-dose Pb-treated and As-treated groups exhibited a remarkable alteration in the expression levels of *syn2a*, *gap43*, *elavl3* and *crh* and *shha*, *syn2a*, *pcdh18b*, *elavl3*, *ache*, *crh* and *acth*, respectively. And the low-dose mixed exposure group modified the transcript levels of *shha*, *gfap*, *syn2a*, *pcdh18b*, *gap43*, *elavl3*, *ache*, *manf*, *nr4a2b*, *crh* and *acth*. Notably, the co-exposure group showed more enhanced expression levels of the genes *shha*, *gap43* and crh and more reduced expression of *gfap*, *pcdh18b*, *ache*, *manf*, *nr4a2b* and *acth* than the mono-exposure group. Within the high-dose treatment group, exposure to As alone induced significant changes in *shha*, *syn2a*, *pcdh18b*, *gap43*, *elavl3*, *ache*, *nr4a2b*, *crh* and *acth*. At the same time, exposure to Pb alone and mixed exposure caused significant changes in *shha*, *gfap*, *syn2a*, *pcdh18b*, *gap43*, *elavl3*, *ache*, *manf*, *nr4a2b*, *crh* and *acth*. A more enhanced expression level of the genes *shha*, *gap43* and *crh* was shown in the joint group in comparison with the single exposure group, while the expression of *gfap*, *syn2a*, *pcdh18b*, *ache*, *manf*, *nr4a2b* and *acth* was more reduced. Furthermore, compared with the low-dose combined exposure group, the high-dose treatment resulted in altered gene expression levels of *shha*, *gfap*, *syn2a*, *gap43*, *manf*, *crh* and *acth*.

In male brains, single low concentrations of Pb or As resulted in significant changes in the genes *gfap*, *syn2a*, *gap43*, *elavl3*, *ache*, *manf*, *nr4a2b* as well as *shha*, *syn2a*, *pcdh18b*, *gap43*, *elavl3*, *ache*, *manf*, *nr4a2b*, respectively. Meanwhile, transcript levels of *shha*, *gfap*, *syn2a*, *pcdh18b*, *gap43*, *elavl3*, *ache*, *manf*, *nr4a2b*, *crh* and *acth* were altered in the low-dose mixed exposure group. The co-exposure group showed more enhanced expression levels of genes *gap43* and *crh,* whereas the expression of *syn2a*, *gfap*, *pcdh18b*, *ache*, *nr4a2b* and *acth* was found to be more reduced than in the single-exposure group. Among the high-dose treatment groups, high doses of Pb or As, respectively, generated notable variations in the transcript levels of genes apart from *ache* and *crh*. The combination exposure groups varied the transcript levels of *shha*, *gfap*, *syn2a*, *pcdh18b*, *gap43*, *elavl3*, *ache*, *manf*, *nr4a2b*, *crh* and *acth*. More precisely, the expression levels of *gfap*, *syn2a*, *pcdh18b*, *gap43*, *elavl3*, *manf*, *nr4a2b*, *crh* and *acth* differed significantly between the combined exposure group and the separate exposure group. Furthermore, the high-dose treatment resulted in a greater decrease in the expression levels of *syn2a* and *manf* compared to the low-dose combined exposure group.

## 4. Discussion

In the present study, zebrafish were separately exposed to Pb and As, or Pb + As, for 30 days. Our results indicated that both Pb and As could lead to neurotoxic effects in zebrafish with no significant difference in gender. However, compared with a single exposure, the co-exposure of Pb and As could exacerbate the toxic effects on the nervous system of zebrafish by altering AChE activity, DA, 5-HT, Cor and EPI levels in the zebrafish brain, and the transcriptional levels of genes associated with neuronal development. Our findings suggest the joint toxic effects of heavy metals on fish in natural water bodies should attract attention.

The swimming behavior of zebrafish was demonstrated to be an important indicator for detecting environmental chemical neurotoxicity [46]. Locomotor behavior is sensitive to many environmental contaminants [47,48]. In the present study, we examined the zebrafish swimming speed, and the results showed that exposure to Pb or As could interfere with swimming speed. Further investigations showed that combined exposure could exacerbate abnormal swimming behavior. Previous studies revealed that joint exposure to Pb and As caused increased anxiety behavior, learning memory and other neurobehavioral abnormalities in mice compared to individually treated groups, which directly affected brain function accordingly [49]. In addition, after chronic low-dose exposure to As, zebrafish displayed enhanced anxiety behaviors with disruption of the neurotransmitter system [50]. In conclusion, the significant reduction in the swimming speed of zebrafish in the combined exposure group in our study may indicate a synergistic effect of As and Pb in generating neurotoxicity.

Behavioral abnormality in zebrafish has long been implicated in brain damage [51]. In our study, histopathological brain damage (the formation of glial scarring and ventriculomegaly) was identified for both genders after 30 days of exposure. Moreover, combined exposure can cause more extensive damage. Ortiz et al. found that anthropogenic damage to zebrafish brain tissue may alter their escape behavior [52]. Liao et al. found that neuronal damage was observed in the brains of zebrafish after radiation, which eventually accounted for the behavioral variation [53]. Similar to these findings, the phenomenon of structural changes in zebrafish brains in this experiment is consistent with the results of abnormal swimming behavior. Thus, our results indicate that As + Pb exposure may trigger neurotoxicity by exacerbating brain damage, thereby leading to increased abnormal swimming behavior.

With the aim of exploring other potential mechanisms of neurotoxicity due to co-exposure to Pb and As, we examined the activity of AChE. Our results indicate that AChE activity was significantly lower. The joint exposure group aggravated this abnormal effect. The cholinergic system is known to be a target of environmental toxins [54]. As part of the cholinergic system, AChE is a critical component in nerve conduction and muscle activity in zebrafish [55]. When ACh is released into the synaptic gap, it is rapidly degraded by AChE [56]. Howeer, alterations of AChE can lead to corresponding changes in ACh levels [57]. Subsequently, altered ACh concentrations may cause impaired muscle contraction and behavioral responses [58]. Accordingly, a probable explanation for the altered swimming behavior in our experiments is a disturbance in the cholinergic system, primarily owing to changes in AChE activity. Due to the fact that gene expression during zebrafish development is a potential marker for rapid screening for neurotoxicity [59], we also tested the gene expression of *ache*. The marked downregulation of *ache* is in accordance with the decrease in AChE activity. From another perspective, the variation in *ache* expression supports our view that changes in the swimming behavior of zebrafish are linked to the dysregulation of the cholinergic system. Interestingly, high levels of Pb and As exposure were proven to significantly alter AChE activity and its neurobehavior in rat brains [14]. This may be due to the interaction of heavy metals with acetylcholine receptors, which affects the efficiency of their binding, giving rise to altered AChE synthesis [49]. Additionally, co-exposure to As and Pb significantly diminished AChE levels, demonstrating that they may work together to intensify disruptions in the cholinergic system, thereby modifying swimming behavior.

In fish, a key factor contributing to heavy metal-induced neurotoxicity is the alteration of neurotransmitter levels [60]. Therefore, we further determined the content of the neurotransmitters DA and 5-HT in zebrafish brains. The results showed that DA and 5-HT concentrations were more decreased in the Pb + As exposure group as compared to the single exposure group. According to the previous literature, malfunctions in the DA and 5-HT systems are strongly involved in neurological health impairments such as movement, memory and attention in organisms [15]. Modified dopaminergic signaling can lead to altered neurobehavior in zebrafish and can facilitate the development of hippocampal neurons [61,62,63]. 5-HT has a key role in participating in neurobehavioral responses [64]. 5-HT for the maturation of swimming patterns in zebrafish larvae is essential and encourages a greater constancy of motor output. Heavy metal exposure was reported to cause aberrant neurotransmitters and their metabolite levels in organisms, ultimately leading to hypoactivity [65,66,67,68], which is consistent with our results. Consequently, the decreased neurotransmitter levels in our study were an influential factor in altering the neurobehavior of zebrafish. A possible reason for this is that the entrance of metal ions into the zebrafish interferes with the correct secretion of neurotransmitter nerves and their ability to bind properly to neurotransmitters receptors, intensified by the simultaneous exposure to two metal ions [69,70]. To further explore the molecular mechanisms, we tested the expression levels of *manf* and *nr4a2b*, genes concerned with DA levels. We found that the transcript levels of these genes were downregulated after exposure. *Manf* is a dopaminergic neurotrophic factor with primarily neural protective properties [71]. It can protect dopaminergic neurons from neurotoxic damage [72]. The *nr4a2b* is also critical for the differentiation, maturation and survival of DA precursor cells during early zebrafish embryonic development [73]. Taken together, the reduced DA levels in brains may relate to the downregulation of the *manf* and *nr4a2b*, which may eventually lead to altered neurobehavior in zebrafish. In summary, our study found that Pb + As may further alter the swimming behavior of zebrafish by interfering with the DA and 5-HT signaling systems.

Another major factor that may determine the development of neurotoxicity after heavy metal exposure is the dysfunction of the HPI axis. The triggering of the HPI axis and the ensuing increase in the secretion of related hormones leads to a comprehensive response, including neurological, endocrine and immune responses, which is an integral feature of the toxic effects [74]. Cor and EPI are hormones that are part of the HPI axis and regulate metabolism, cognitive function and anxiety [75]. Indeed, it was proposed that the stress response to heavy metal-induced neurotoxicity is connected to elevated levels of catecholamines and Cor [76,77]. In this study, we observed that Pb or As enhanced HPI axis activity, causing elevated transcript levels of the *crh* gene and ultimately leading to significantly higher Cor and lower EPI levels. Pb + As augmented the effects of single metal exposures on the HPI axis. This is in accordance with several earlier studies that revealed heavy metal exposure can elevate Cor contents and contribute to the permanent dysfunction of the HPI axis, eventually displaying more anxious behavior and triggering neurotoxicity [16,78,79]. A possible explanation is that excessive Cor secretion feeds back to inhibit the hypothalamic–pituitary axis, causing a decrease in adrenocorticotropic hormone (ACTH) release, which shrinks the adrenal cortex and, thereby, diminishes adrenocortical function [80]. This idea is supported by the downregulation of the *acth* gene in the results. Consequently, it is evident that Pb + As further activates the HPI axis by impairing both Crh and EPI levels, subsequently leading to aberrant swimming behavior.

Apart from changes in neurotransmitter levels and HPI axis function, abnormal expression of central nervous system (CNS) related genes may also be a generational effect of neurotoxicity induced by Pb and As. CNS is the handling heart of the nervous system, where it performs as the principal agent in the regulation of zebrafish locomotion [69]. Our study examined the expression of several CNS-related genes, including *shha*, *gfap*, *syn2a*, *pcdh18b*, *gap43* and *elavl3*. Of these genes, *shha* and *gap43* were significantly upregulated following exposure, while gene expression was significantly upregulated after exposure to Pb + As. The *shha* gene manages events surrounding neural stem cell proliferation and neuronal and glial cell survival as an integral marker for rapid screening of neurotoxic effects [81,82]. The *gap43* gene is thought to be a marker for the re-induction of axonal growth after injury to promote regeneration [83]. The increased expression of the *gap43* protein has been reported as necessary to counteract direct damage from toxicants [84]. Accordingly, the overexpression of *shha* and *gap43* may be an adaptive response and possibly a compensatory response, suggesting that exposure to Pb + As stimulates the initiation of brain damage repair mechanisms. Keeping cells safe from neurotoxic interference together with co-exposure promotes this protective mechanism. Alternatively, transcript levels of *gfap*, *syn2a*, *pcdh18b* and *elavl3* were significantly downregulated following Pb and As exposure. In addition, the inhibitory effect of combined exposure on gene expression was more pronounced. The *gfap* is a constituent marker of astrocytes and is involved in the regulation of the blood–brain barrier, as well as in various pivotal central nervous system processes [85]. The expression of *gfap* was downregulated, suggesting that Pb + As exposure can lead to glial cell damage and inhibit the development and maturation of the nervous system. *Syn2a*, a neuronal phosphoprotein, induces prominent formation in mammals and plays an important role in neurotransmitter release [86]. The abnormal expression of *syn2a* and *shha* suggests a joint effect of Pb + As exposure on neural signaling and neuronal myelin formation. The *pcdh18b* gene, broadly expressed in the central nervous system, is involved in the development of nerve cells and the correct migratory localization of cells in the border region of the mid- and hindbrain [87]. Actually, Pb + As may have an impact on the nervous system by affecting the correct migration and development of neuronal cells in zebrafish. *elavl3* encodes a neurospecific RNA-binding protein that plays an invaluable part in neuronal development and individual behavior [88]. The altered expression of the *elavl3* implies that heavy metal exposure can induce abnormal neuronal growth. On the basis of these observations, it is reasonable to speculate that Pb + As exposure causes neurological damage in the zebrafish brain, affecting synaptogenesis, cytoskeletal regulation, neurotransmitter release, neuronal maturation and, ultimately, neurotoxicity.

## 5. Conclusions

In summary, both individual and combined exposure to the heavy metals Pb or As may contribute to neurotoxicity in zebrafish. The changes in the swimming behavior of zebrafish were abnormally aggravated in the co-exposure group. Another aspect reveals that the neurotoxicity caused by Pb + As in zebrafish may be synergistic, which exacerbates the single metal toxicity. Moreover, we found that the neurotoxicity caused by Pb + As may be a result of a combination of heightened levels of brain damage, alterations in the cholinergic system, effects of DA and 5-HT signaling systems and the dysfunction of the HPI axis and neuronal development. This study not only provides a theoretical basis for the ecological risk assessment of Pb and As but also further emphasizes the importance of considering the interaction of heavy metals at environmentally relevant concentrations and provides sufficient motivation for regulatory agencies to reconsider the health risks of chemical mixtures.

## Data Availability

The data presented in this study are available on request from the corresponding authors.

## Supplementary Materials

The following supporting information can be downloaded at: https://www.mdpi.com/article/10.3390/toxics12040282/s1, Table S1: Primer sequences used for qRT-PCR; Table S2: The temperature, pH and ammonia nitrogen content in the experimental water before and after water renewed (n = 3); Table S3: The concentrations of fluorescent Pb and As in the exposed water before and after water renewed (n = 3).
