# Supplementary material for: Neurotoxicity of Combined Exposure to the Heavy Metals (Pb and As) in Zebrafish (Danio rerio)
Source: Toxics. 2024 Apr 11;12(4):282. doi: 10.3390/toxics12040282 (PMC11054020; doi:10.3390/toxics12040282)
Supplement: Supplementary file 1 [file toxics-12-00282-s001.zip › toxics-2945909-supplementary.pdf]

Neurotoxicity of Combined Exposure to the Heavy Metals （Pb and As） in Zebrafish (*Danio rerio*)

Ming Liu[1#], Ping Deng[3#], Guangyu Li[1], Haoling Liu[1], Junli Zuo[1], Wenwen Cui[2], Huixian Zhang[2], Xin Chen[2], Jingjing Yao[2], Xitian Peng[2], Lijun Peng[2], Jiao Liu[2], Wenting Zheng[1], Wei Yan[2*], Ning Luan[1*]

[1]College of Fisheries, Huazhong Agricultural University, Wuhan, 430070, China

[2]Institute of Quality Standard and Testing Technology for Agro-Products, Hubei Academy of Agricultural Sciences, Hubei Key Laboratory of Nutritional Quality and Safety of Agro-products, Wuhan, 430064, Hubei, China

[3]Study and practical demonstratiministry on regime shifts and optimization of ecosystem after ecological restoration project 'turning fishpond to wetland' in Chenhu Lake, Wuhan Academy of Agricultural Sciences, Wuhan, 430056, China

*Corresponding Author:

Ning Luan (N. Luan)

College of Fisheries,

Huazhong Agricultural University,

Wuhan 430070, China

Fax: 86-27-87282114

Email: luanning@webmail.hzau.edu.cn

Wei Yan (W. Yan) PhD

Hubei Key Laboratory of Nutritional Quality and Safety of Agro-products,

Hubei Academy of Agricultural Sciences,

Wuhan 430064, China

Fax: 027-87389465

Email: yanwei75126@163.com

[#]These two authors contribute equally to this work

[*]These two authors contribute equally to this work

Table S1. Primer sequences used for qRT-PCR.

Gene name	Primer sequences (from 5′ to 3′)
syn2a	F: GTGACCATGCCAGCATTTC
	R: TGGTTCTCCACTTTCACCTT
pcd18b	F: GTTGTTCCCATATTTGAAGACGTGC
	R: CCTCTTACCTCAGTTACAATTTATA
shha	F: GCAAGATAACGCGCAATTCGGAGA
	R: TGCATCTCTGTGTCATGAGCCTGT
gfap	F: GGATGCAGCCAATCGTAAT
	R: TTCCAGGTCACAGGTCAG
acth	F: AATTTGCACTTGCCTATG
	R: CCCTTTGTAGCGTCATT
crh	F: TTCGGGAAGTAACCACAAGC
	R: CTGCACTCTATTCGCCTTCC
manf	F:AGATGGAGAGTGTGAAGTCTGTGTG
	R: CAATTGAGTCGCTGTCAAAACTTG
ache	F:CCCTCCAGTGGGTACAAGAA
	R: GGGCCTCATCAAAGGTAACA
gap43	F: TGCTGCATCAGAAGAACTAA
	R:CCTCCGGTTTGATTCCATC
β-actin	F: ATGGATGAGGAAATCGCTGCC
	R: CTCCCTGATGTCTGGGTCGTC
elavl3	F:AGACAAGATCACAGGCCAGAGCTT
	R:TGGTCTGCAGTTTGAGACCGTTGA
nr4a2b	F:GAAGACGGCGAAATCGATGC
	R:CTGGCGGTTCTGACAACTTCC

Table S2. The temperature, pH and ammonia nitrogen content in the experimental water before and after water renewed (n = 3).

	Before	After
Ammonia nitrogen content (mg/L)	0.7278±0.0907	0.395±0.046
pH	6.76±0.041	7.18±0.031
T (°C)	24.7±0.2	24.5±0.1

Table S3. The concentrations of fluorescent Pb and As in the exposed water before and after water renewed (n = 3).

Groups	Before		After	
	As (mg/L)	Pb (mg/L)	As (mg/L)	Pb (mg/L)
0	-	-	-	-
Low-Pb	-	0.0194±0.0009	-	0.0304±0.0025
Low-As	0.0191±0.0013	-	0.036±0.0075	-
Low-Pb+As	0.0229±0.0014	0.0207±0.0024	0.0336±0.0019	0.0296±0.0004
High-Pb	-	3.0423±0.0681	-	3.7674±0.2688
High-As	2.4667±0.0973	-	2.9967±0.1889	-
High-Pb+As	2.19±0.0424	3.1147±0.0471	3.3133±0.2518	3.9864±0.0215
